# Distribution of interscan measurement error in AI-based 3D MRI analysis of knee cartilage thickness in osteoarthritis

**DOI:** 10.1371/journal.pone.0329610

**Published:** 2025-08-07

**Authors:** Hisako Katano, Eiji Sasaki, Kanto Nagai, Naofumi Hashiguchi, Haruka Kaneko, Yasuyuki Ishibashi, Ryosuke Kuroda, Nobuo Adachi, Muneaki Ishijima, Makoto Tomita, Jun Masumoto, Ichiro Sekiya

**Affiliations:** 1 Center for Stem Cell and Regenerative Medicine, Institute of Science Tokyo, Tokyo, Japan; 2 Department of Orthopaedic Surgery, Hirosaki University Graduate School of Medicine, Aomori, Japan; 3 Department of Orthopaedic Surgery, Kobe University Graduate School of Medicine, Hyogo, Japan; 4 Department of Orthopaedic Surgery, Graduate School of Biomedical and Health Sciences, Hiroshima University, Hiroshima, Japan; 5 Department of Medicine for Orthopedics and Motor Organ, Juntendo University Graduate School of Medicine, Tokyo, Japan; 6 School of Data Science, Graduate School of Data Science, Yokohama City University, Kanagawa, Japan; 7 Fujifilm Corporation, Tokyo, Japan; Mie University Graduate School of Medicine, JAPAN

## Abstract

**Purpose:**

A novel AI-based 3D analysis system was developed to automatically extract bone and cartilage from MRI data and provide average cartilage thickness. This study aimed to analyze the interscan measurement error of knee cartilage thickness in osteoarthritis patients.

**Methods:**

Fifty knee osteoarthritis patients underwent two scans using MRI systems from five different vendors. Each model included five Kellgren-Lawrence grade (KL) 1–2 and five KL3–4 patients. Cartilage thickness was automatically quantified for seven regions, and interscan measurement error was analyzed.

**Results:**

In the KL1–2 group, measurements with errors ≤0.05 mm, ≤ 0.10 mm, and ≤0.20 mm were 42%, 75%, and 97%, respectively. For the KL3–4 group, these proportions were 31%, 59%, and 90%. The entire cohort (KL1–4) showed errors ≤0.05 mm, ≤ 0.10 mm, and ≤0.20 mm in 37%, 67%, and 93% of measurements. Differences between KL1–2 and KL3–4 groups were significant for all thresholds.

**Conclusion:**

Overall, 93% of interscan measurement errors were within 0.20 mm when using fully automatic MRI 3D analysis software to assess knee cartilage thickness in osteoarthritis patients. This study provides valuable insights into the reliability of automated cartilage thickness measurements across different disease severities and MRI systems.

## Introduction

Osteoarthritis of the knee, the most common knee disorder, causes pain and functional impairment [1]. Evaluation of the progression of this disease and determination of the effectiveness of disease-modifying osteoarthritis drugs require an accurate assessment of the condition of the knee cartilage [2]. In recent years, non-invasive evaluation methods using magnetic resonance imaging (MRI) have been attracting attention.

The three-dimensional analysis system developed in this study is software that fully automatically extracts bone, cartilage, and meniscus from 2D MRI data using AI and generates 3D images [3]. This software enables rapid and reproducible measurement of the average cartilage thickness in the region of interest [4]. Previous studies have reported the interscan measurement error in cartilage thickness obtained using healthy volunteers [5]. Recent research has also confirmed no difference in the quantitative value of cartilage thickness when the same knee was imaged using instruments from five different MRI manufacturers [6].

The interscan measurement error of cartilage thickness in this system was unknown for patients with knee osteoarthritis. Since osteoarthritis leads to cartilage degeneration and reduces cartilage thickness, measurement errors may differ from those obtained for healthy individuals. Therefore, the purpose of this study was to analyze the interscan measurement error of knee cartilage thickness in patients with knee osteoarthritis. To better fit clinical practice, we used data obtained using MRI equipment manufactured by five different companies (Canon, Fujifilm, Philips Healthcare, Siemens, and GE Medical Systems).

## Subjects and methods

### Subjects

This study was approved by the Institutional Review Board of the Institute of Science Tokyo (approval number: M2021-291). All participants provided written informed consent before enrollment. The study included 50 patients with medial knee osteoarthritis, with only unilateral knees examined per patient. The cohort was predominantly female (66%) with an age range of 29–85 years and a median age of 59 years (Table 1). MRI scans were performed between September 13, 2022, and April 3, 2024. All measurements were conducted on the right knee of each participant.

**Table 1:** Subject population demographics.

		Canon		Fujifilm		GE		Philips		Siemens		Total
		KL1–2	KL3–4	KL1–2	KL3–4	KL1–2	KL3–4	KL1–2	KL3–4	KL1–2	KL3–4	
Gender	Female	2	1	3	5	3	4	2	5	5	3	33
	Male	3	4	2	0	2	1	3	0	0	2	17
	Female ratio	0.40	0.20	0.60	0.50	0.60	0.80	0.40	1.00	1.00	0.60	0.66
Age	Median	61	52	76	76	46	57	54	74	45	69	59
	Maximum	65	60	82	85	63	81	62	82	57	73	85
	Minimum	53	32	58	67	31	50	45	54	29	66	29

### Kellgren–Lawrence (KL) grade

Radiographs were taken of the anteroposterior view of the knee, with the patient in a standing position with full knee extension [7; 8]. Medial knee osteoarthritis was graded according to the modified KL grade described by Guermazi et al. [9]. KL grade 0 shows no features of osteoarthritis. KL grade 1 shows osteophytic lipping (equivocal osteophytes). KL grade 2 shows definite (unequivocal) osteophytes. KL grade 3 shows joint space narrowing. KL grade 4 shows definite deformity of the bone ends (bone-to-bone appearance) [10]. Five KL1–2 subjects and five KL3–4 subjects were included for evaluation using each of the five different MRI machines.

### MRI protocol

**Fig 1:**
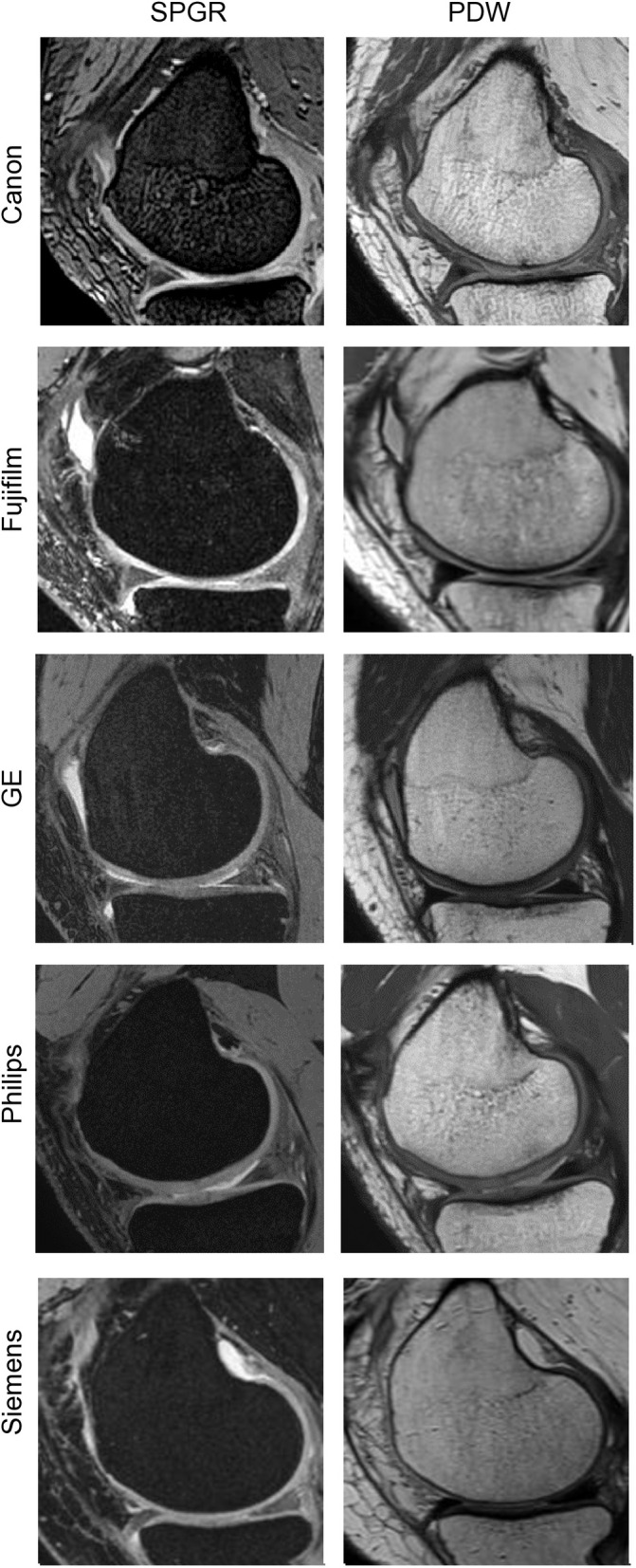
Sagittal MR images of the knee taken with MRI machines from five different companies. Representative examples were selected from each company.

The study employed five distinct 3T MRI systems from various manufacturers, each located in different cities across Japan. These included devices from Canon, Fujifilm, Philips Healthcare, Siemens, and GE Medical Systems. Imaging was performed on the sagittal plane using two protocols: a fat-suppressed spoiled gradient echo (SPGR) sequence and a proton density-weighted (PDW) sequence (Fig 1). Prior to the main study, a preliminary test was conducted to optimize the sequence parameters for articular cartilage visualization on each system. The resulting optimal settings are presented in Table 2. The scanning procedure involved two consecutive MRI sessions for each participant. After the initial scan, subjects were asked to stand and then reposition themselves for a second scan. This approach allowed for assessment of the reproducibility of measurements across different scanning sessions.

**Table 2:** Imaging parameters for the MRI sequences.

Company	Canon	Fujifilm	GE	Philips	Siemens
Model Name	Centurian	TRILLIUM_OVAL	SIGNA Premier	Ingenia	Skyra
Receive Coil Name	16chFlex SPDR M	Extremity	18Knee	SENSE_KNEE_16_AC	#N/A
Transmit Coil Name	QD Whole Body	#N/A	18Knee	BODY	TxRx_Knee_15
**SPGR**					
Slice Thickness (mm)	0.70	0.80	0.60	0.60	0.36
Repetition Time (ms)	20.80	8.40	11.30	23.19	13.46
Echo Time (ms)	11.40	4.20	4.90	6.91	5.00
Echo Number(s)	1.00	1.00	1.00	1.00	1.00
Magnetic Field Strength (T)	3.00	2.90	3.00	3.00	3.00
Spacing Between Slices (mm)	0.35	0.40	0.30	0.30	#N/A
Number of Phase-Encoding Steps	136	256	#N/A	254	223
Echo Train Length	1	0	1	3	2
Percent Phase Field of View (%)	100	100	100	100	100
Pixel Bandwidth (Hz)	279	#N/A	98	310	285
Acquisition Matrix (Frequency Row/ Frequency Column/ Phase Row/ Phase Column)	0/272/272/0	0/256/256/0	0/320/320/0	0/256/254/0	0/256/256/0
Pixel Spacing (mm)	0.29/0.29	0.31/0.31	0.31/0.31	0.29/0.29	0.29/0.29
Flip Angle (degree)	4	20	25	30	15
SAR (w/kg)	0.56	0.13	0.03	0.01	0.01
dB/dt (T/s)	8.59	0.67	#N/A	96.33	0.00
					
**PDW**					
Slice Thickness (mm)	0.70	0.80	0.59	0.60	0.36
Repetition Time (ms)	1100	1500	1000	1000	1000
Echo Time (ms)	13.00	34.40	31.65	31.47	34.00
Echo Number(s)	1.00	1.00	1.00	1.00	1.00
Magnetic Field Strength (T)	3.00	2.90	3.00	3.00	3.00
Spacing Between Slices (mm)	0.35	0.40	0.30	0.30	#N/A
Number of Phase-Encoding Steps	80	224	#N/A	254	123
Echo Train Length	18	38	60	40	24
Percent Phase Field of View (%)	100	100	100	100	100
Pixel Bandwidth (Hz)	488	#N/A	244	592	330
Acquisition Matrix (Frequency Row/ Frequency Column/ Phase Row/ Phase Column)	0/320/320/0	0/224/224/0	0/320/320/0	0/256/254/0	0/320/208/0
Pixel Spacing (mm)	0.25/0.25	0.33/0.33	0.31/0.31	0.29/0.29	0.23/0.23
Flip Angle (degree)	90	90	90	90	120
SAR (w/kg)	0.64	0.65	0.26	0.04	0.05
dB/dt (T/s)	13.96	0.72	#N/A	87.31	0.00

SPGR = fat-suppressed spoiled gradient echo; PDW = proton density weighted; #N/A = not applicable; SAR = specific absorption rate; dB/dt = rate of change of the magnetic field.

### Cartilage thickness evaluation protocol

**Fig 2:**
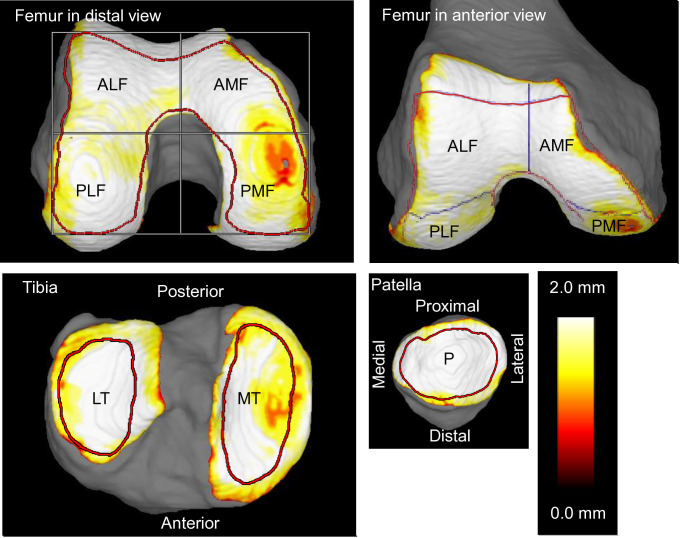
MRI cartilage thickness mapping and regions of the knee. The region of interest is indicated by a red line, and the femoral cartilage is divided into four regions by halving it vertically and horizontally in the distal view. PMF, posterior medial femoral; PLF, posterior lateral femoral; AMF, anterior medial femoral; ALF, anterior lateral femoral; MT, medial tibial; LT, lateral tibial; P, patellar.

The study utilized 3D volume analysis software for MRI processing, SYNAPSE 3D version 6.8 (known as SYNAPSE VINCENT in Japan, Fujifilm Corporation, Tokyo, Japan). This software employs deep-learning algorithms for image segmentation. Three-dimensional bone images served as the foundation for identifying the cartilage regions of interest (ROIs). These ROIs were defined by automatic determination of the bone’s morphology and surface characteristics. The automatic segmentation process operates using pre-configured deep-learning algorithms that have been trained and optimized specifically for cartilage analysis, requiring no manual adjustment during the segmentation procedure. This automatic segmentation algorithm has demonstrated high accuracy with Dice similarity coefficients (segmentation accuracy index: 0–1, where 1 = perfect match) of 0.98–0.99 for femoral and tibial bone, and 0.89–0.91 for femoral cartilage, tibial cartilage, ROI of femoral subchondral bone, and ROI of medial/lateral tibia plateau [3]. For cartilage thickness measurements, areas 2 mm inward from the initially defined cartilage region were designated as the final ROIs (Fig 2).

For analysis of the femoral cartilage, a projection technique along the femur’s longitudinal axis was utilized. To standardize the rotation, a horizontal reference line connecting the posteromedial and posterolateral cartilage ROIs was established. The analysis software then generated perpendicular lines, dividing the ROI into four equal quadrants along both the long and short axes. Similarly, the tibial cartilage was projected along the tibia’s main axis. The software automatically delineated separate ROIs for the medial and lateral tibial plateaus, represented as two enclosed contours [11]. For the patellar cartilage, the approach involved maximizing the visible cartilage area in the projection. This resulted in an automatically defined ROI, outlined as a single closed curve [12].

To visualize cartilage thickness, a color-coded mapping system was employed. This representation utilized a spectrum where white denoted the thickest regions and red signified the thinnest areas. The analysis software divided the cartilage layer immediately above the bone’s surface into the smallest measurable segments. It then calculated the thickness of each individual segment and computed the mean cartilage thickness for the entire ROI.

### Evaluating cartilage thickness measurement variability between scans

The interscan measurement error of cartilage thickness was defined as the absolute difference between the first and second thickness measurements. To visualize the distribution of these errors, histograms were constructed for three groups: KL1–2 (comprising 175 measurements from 5 subjects, 7 regions, and 5 MRI systems), KL3–4 (also 175 measurements), and the entire cohort (KL1–4) (totaling 350 measurements). The percentage of measurements falling within three error thresholds was then calculated: ≤ 0.05 mm, ≤ 0.10 mm, and ≤0.20 mm.

### Relationship between cartilage thickness and interscan measurement error

The cartilage thickness for each measurement was calculated as the average of the measurements made at the first and second time points. The cartilage thickness measurements were divided into the following four groups for analysis: 0–1 mm, greater than 1 mm to 2 mm, greater than 2 mm to 3 mm, and greater than 3 mm to 4 mm.

## Data analysis

GraphPad Prism 10 (GraphPad Software, Boston, MA, USA) was used to generate individual cartilage thickness plots by subject and region, while Microsoft Excel 2021 (Microsoft, Redmond, WA, USA) was used to construct histograms of interscan measurement error distributions. Cartilage thickness between the KL1–2 and KL3–4 groups at each anatomical region was compared using the Mann–Whitney U test. To evaluate differences in the frequency of interscan measurement errors falling within three predefined thresholds (≤0.05 mm, ≤ 0.10 mm, and ≤0.20 mm), chi-square tests were conducted. To assess whether the interscan measurement error decreased with decreasing cartilage thickness, the Jonckheere–Terpstra test was used to evaluate trends across the following four thickness-based subgroups. Statistical analyses were performed using GraphPad Prism 10 and the BellCurve extension for Excel (Social Survey Research Information Co., Ltd., Tokyo, Japan), with significance defined as p < 0.05.

## Results

### Cartilage thickness

**Fig 3:**
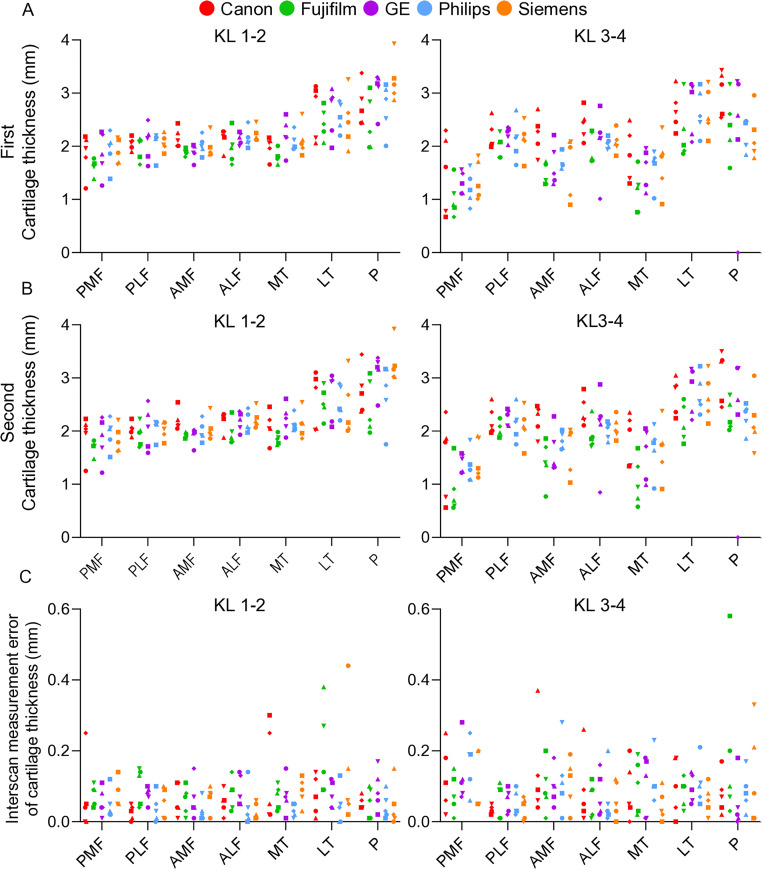
Cartilage thickness and interscan measurement errors at seven regions for 25 subjects in KL1-2 and 25 subjects in KL3-4. **(A)** Cartilage thickness measured at the first time point. Five subjects from KL1-2 and five subjects from KL3-4 for each MRI system from five different vendors are plotted in the corporate colors. **(B)** Cartilage thickness measured at the second time point. The same symbol is shown for the same subject measured at the first time point. **(C)** Interscan measurement error. The absolute value of the difference between the cartilage thickness measured at the first time point and the second time point is shown.

**Fig 4:**
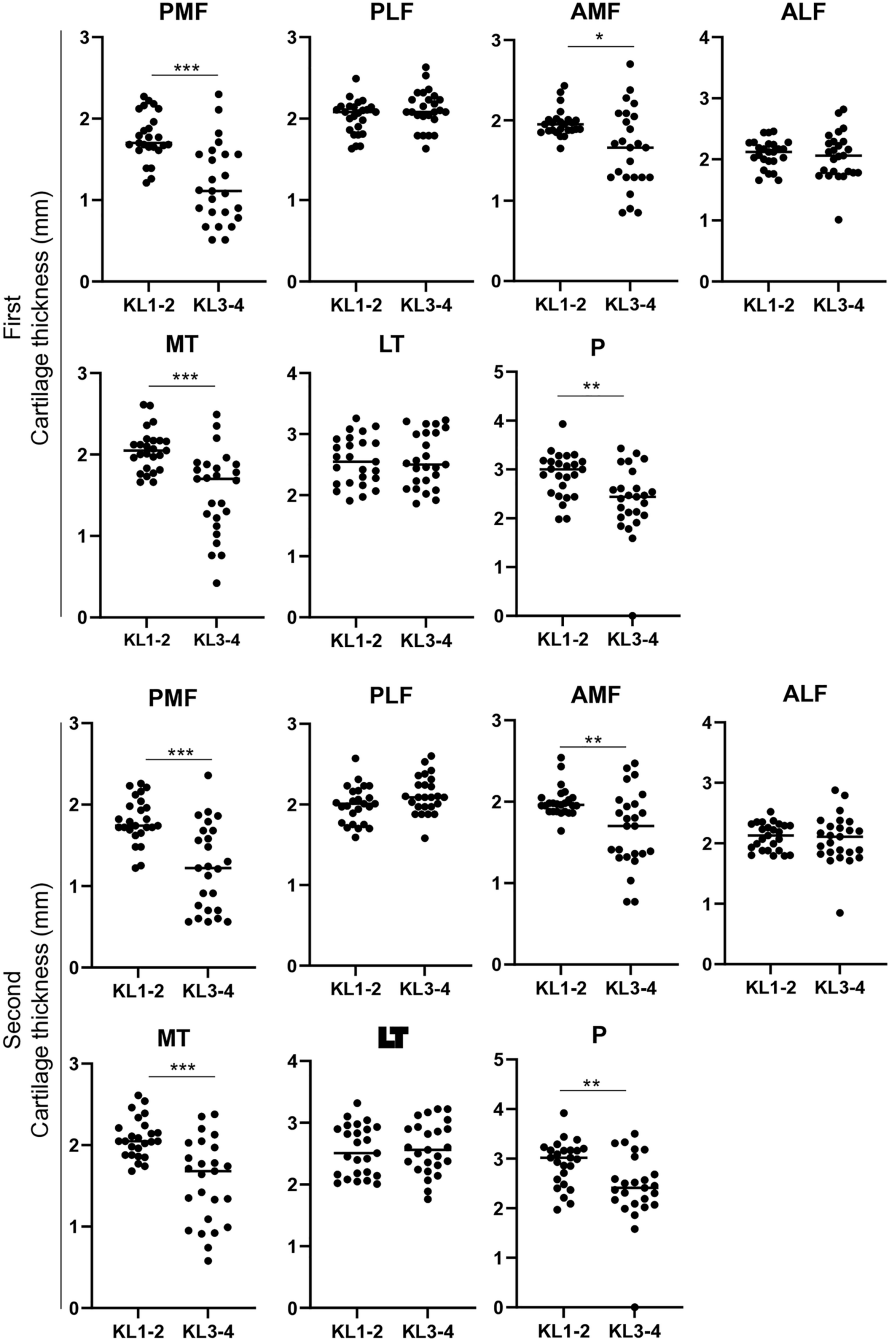
Comparison of cartilage thickness between the KL1-2 and KL3-4 groups. The median is indicated by a horizontal line. *, p < 0.05 by Mann-Whitney U test (n = 25).

Cartilage thickness was measured at 7 regions for 5 subjects from KL1–2 and 5 subjects from KL3–4 on MRI models using MRI scans from 5 different vendors and plotted (Fig 3A). Subjects stood up once and were scanned again for the second time, and cartilage thickness was measured and plotted in the same manner (Fig 3B). Comparison of the cartilage thickness between KL1–2 and KL3–4 revealed significantly lower values in KL3–4 than in KL1–2 in the posterior medial femoral (PMF), anterior medial femoral (AMF), medial tibial (MT), and patellar (P) regions for measurements made at both the first and second times (Fig 4).

### Interscan measurement error of cartilage thickness

Fig 3C shows the interscan measurement error, which is the absolute value of the difference between the cartilage thicknesses obtained from the first and second measurements. Interscan measurement errors exceeding 0.2 mm accounted for 6 out of 175 measurements for KL1–2 and 16 out of 175 measurements for KL3–4.

### Distribution of the interscan measurement error

**Fig 5:**
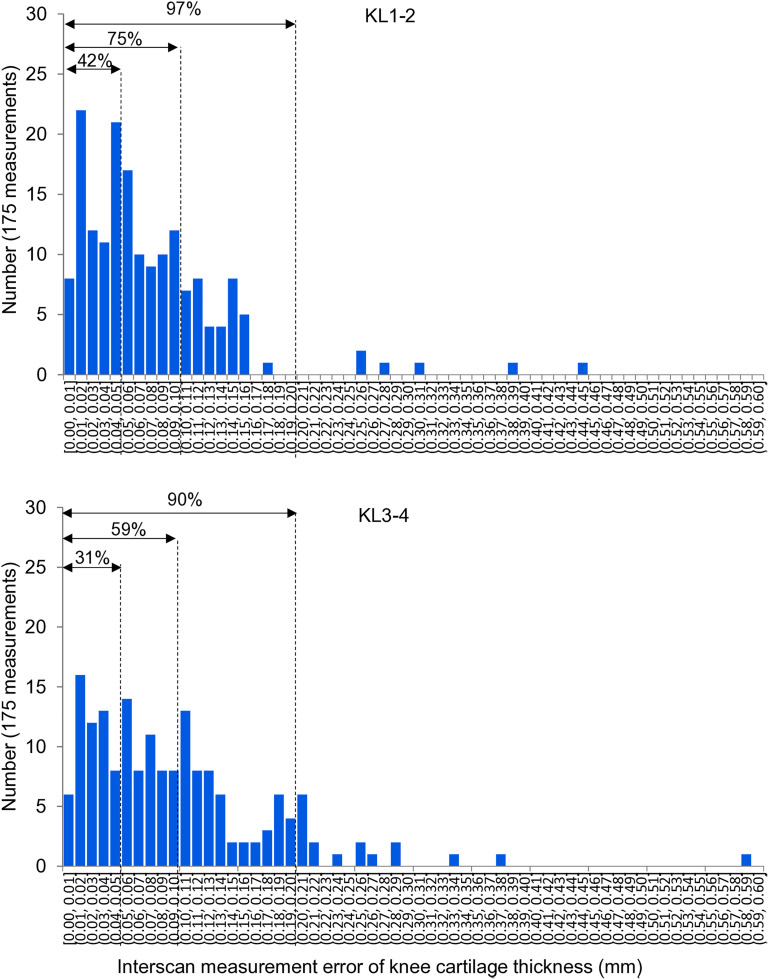
Distribution of the interscan measurement error in cartilage thickness in the KL1-2 and KL3-4 groups. For example, an interscan measurement error of “(0.04, 0.05) mm” indicates 0.04 mm < interscan measurement error ≤ 0.05 mm.

In KL1–2, from a total of 175 measurements, 42% had an interscan measurement error ≤ 0.05 mm, 75% had an interscan measurement error ≤ 0.10 mm, and 97% had an interscan measurement error ≤ 0.20 mm (Fig 5). In KL3–4, 31% had an interscan measurement error ≤ 0.05 mm, 59% had an interscan measurement error ≤ 0.10 mm, and 90% had an interscan measurement error ≤ 0.20 mm. There was a significant difference between the KL1–2 and KL3–4 groups regarding the proportion within 0.05 mm (p = 0.035), 0.10 mm (p = 0.001), and 0.20 mm (p = 0.0001).

**Fig 6:**
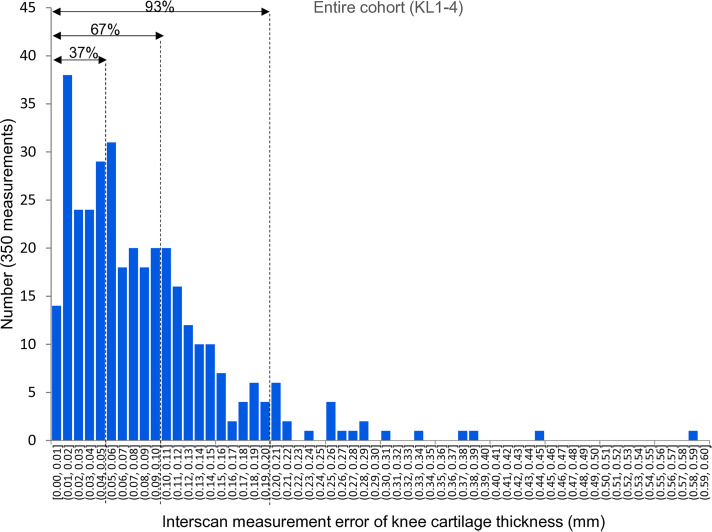
Distribution of the interscan measurement error in cartilage thickness in the entire cohort (KL1-4). Of the total 350 measurements, the interscan measurement error was ≤ 0.05 mm in 37%, ≤ 0.10 mm in 67%, and ≤ 0.20 mm in 93%.

In the entire cohort (KL1–4), from a total of 350 measurements, 37% had an interscan measurement error ≤ 0.05 mm, 67% had an interscan measurement error ≤ 0.10 mm, and 93% had an interscan measurement error ≤ 0.20 mm (Fig 6).

### Cartilage thickness and interscan measurement error

**Fig 7:**
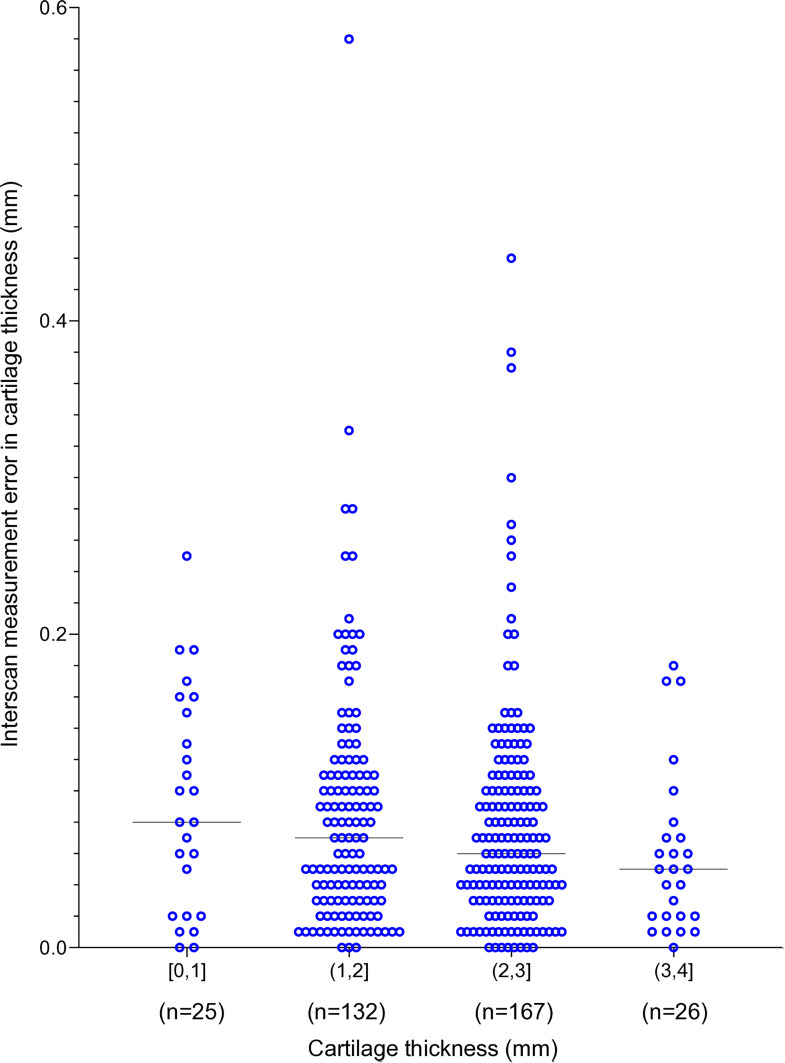
The cartilage thickness and interscan measurement error in cartilage thickness in the entire cohort (KL1–4). The cartilage thickness was calculated as the average of measurements made at the first time point and at the second time point. For example, a cartilage thickness of “(1, 2) mm” indicates a cartilage thickness greater than 1 mm but less than or equal to 2 mm. The medians are indicated by the horizontal lines.

The cartilage measurements were categorized into four thickness groups at 1 mm intervals. The interscan measurement error showed a decreasing trend with increasing cartilage thickness (Fig 7), although the differences were not statistically significant according to the Jonckheere–Terpstra test.

## Discussion

This study analyzed the interscan measurement error of knee cartilage thickness in osteoarthritis patients using fully automatic 3D MRI analysis software. Fifty patients with knee osteoarthritis underwent two scans each using MRI systems from five different vendors. The average cartilage thickness was then automatically quantified for seven regions of the knee. Overall, 93% of the interscan measurement errors were within 0.20 mm. This result suggests that the AI-based 3D analysis system is a promising tool for measuring knee cartilage thickness in osteoarthritis patients, as it demonstrates a relatively small variation between scans in most cases.

The measured cartilage thicknesses were significantly lower in the KL3–4 group than in the KL1–2 group in 4 out of 7 regions. Since the subjects in this study had medial osteoarthritis, a less robust cartilage thickness in the medial regions of the femorotibial joint, such as PMF, AMF, and MT, supports the appropriateness of radiographic evaluation. The cartilage thickness was also found to be thinner in the patellar region. At present, no correlation appears to have been definitively established between the joint space width between the medial femorotibial joint and the patellofemoral joint in medial knee osteoarthritis.

The proportion of interscan measurement errors ≤ 0.05 mm, ≤ 0.10 mm, and ≤ 0.20 mm was lower in the KL3–4 group than in the KL1–2 group. This indicates that interscan measurement errors were higher in the KL3–4 group than in the KL1–2 group. This observation was further supported by our analysis, which showed a decreasing trend in interscan measurement error with increasing cartilage thickness. The lack of statistically significant differences was likely a reflection of the uneven sample distribution across the thickness groups. Nevertheless, this trend suggests that thinner cartilage is associated with greater measurement variability. Measuring thin cartilage thickness using MRI is more prone to errors due to three main factors: pixel resolution limitations, partial volume effects, and image noise and contrast issues. Thin cartilage may span only one or a few pixels, making accurate representation difficult. The partial volume effect occurs when a voxel contains multiple tissue types, thereby altering the signal intensity and leading to inaccurate measurements. Noise and low contrast in MRI images also add to the challenge of distinguishing thin cartilage from surrounding tissues, thereby further increasing measurement errors [13,14].

For 9 healthy volunteers with KL0 knees, a previous study used the same system to determine the interscan measurement error of cartilage thickness in 6 regions (the patellar region was excluded from the 7 regions in the present study). The interscan measurement error of cartilage thickness was less than 0.10 mm in all of these regions [5]. In the current study, the proportion of interscan measurement error ≤ 0.10 mm was 75% for KL1–2 and 59% for KL3–4. As discussed above, the interscan measurement error increases with the KL grade due to the decrease in cartilage thickness.

We evaluated errors using thresholds of ≤0.05 mm, ≤ 0.10 mm, and ≤0.20 mm. This choice was based on our previous study involving healthy subjects, in which thresholds of ≤0.10 mm and ≤0.20 mm were used to characterize measurement variability [15]. Our adoption of the same thresholds in the present study enabled us to make direct comparisons with those prior findings. Additionally, considering that clinical studies in osteoarthritis may require the detection of very small changes, we included a more stringent threshold of ≤0.05 mm. In the previous study, 82% of the measurements showed variability ≤0.10 mm, and 98% were ≤0.20 mm. In contrast, in the current study involving the entire cohort (KL1–4), 67% and 93% of measurements were within ≤0.10 mm and ≤0.20 mm, respectively. These findings indicate that patients with osteoarthritis may have larger measurement errors, which likely reflect differences in cartilage thickness, as illustrated in Fig 7.

Our sample size of 25 subjects per group (KL1–2 and KL3–4) was based on our previous finding that a similar sample size yielded consistent profiles of interscan variability in healthy subjects [15]. Although no formal power analysis was performed, this sample size (totaling 50 knees) was deemed sufficient for evaluating the distributional characteristics of interscan errors across seven anatomical regions and multiple MRI systems. In total, 350 region-specific measurements (7 regions × 5 knees × 2 KL groups × 5 MRI systems) were included, thereby enhancing the robustness of the variability assessment. This enhancement enabled the visualization of error distributions and regional differences with sufficient granularity, as illustrated in Fig 6.

We categorized the interscan measurement error data into threshold levels (≤0.05 mm, ≤ 0.10 mm, and ≤0.20 mm) rather than analyzing it as a continuous variable for several reasons. First, threshold-based analysis provides clinically meaningful information by indicating the proportion of measurements that fall within acceptable precision limits. Knowing this proportion is directly relevant for clinical decision making and the design of intervention studies. Second, this approach enabled direct comparison with the values found in our previous study of healthy subjects, for which we had used the same threshold methodology [15]. Third, threshold analysis is commonly used in measurement precision studies to establish quality control standards for clinical practice [16]. While continuous analysis could have provided additional statistical information, the threshold approach better addressed the practical question of how often measurements would be sufficiently precise for clinical use, particularly when detecting small changes in cartilage thickness is critical.

The semiautomatic 3D MRI analysis of osteoarthritis knees performed by Eckstein et al. revealed a decrease of 0.18 mm in the cartilage thickness at the central medial femur over 2 years in osteoarthritis knees (KL1–3) with radiographic and pain progression (n = 194) [17]. In the current study, in the entire cohort (KL1–4), the proportion of the interscan measurement error of knee cartilage thickness ≤ 0.17 mm was 89% (=311/350). Thus, the probability that a difference of 0.18 mm is an interscan measurement error was 11%.

The occurrence of interscan measurement error when quantifying knee cartilage thickness using fully automated 3D MRI analysis can be attributed to several factors. Variations in magnetic field homogeneity between scans can affect image contrast and signal intensity, leading to discrepancies in cartilage thickness measurements. The dynamic nature of joint fluid can also cause slight shifts in its distribution, potentially influencing cartilage surface detection and subsequent thickness calculations. Patient movements, even if subtle, during scans can introduce errors in image alignment and segmentation. Moreover, the accuracy of automated algorithms in distinguishing cartilage from surrounding tissues can be challenged, particularly in the presence of structures with similar signal intensities, such as menisci or synovium. Lastly, inconsistencies in defining the region of interest (ROI) between scans can contribute to measurement variations [18; 19].

The pixel spacing of the MRI scans in this study ranged from 0.23 to 0.33 mm, but this variability does not preclude the evaluation of interscan measurement errors smaller than the voxel size. Cartilage thickness was derived from three-dimensional segmentation across multiple contiguous voxels, and subvoxel accuracy was achieved through interpolation and surface-based thickness computation [20]. The thresholds, including the ≤ 0.05 mm threshold, were not intended to reflect absolute spatial resolution but instead served as relative indices of measurement consistency across repeated scans. Previous studies have demonstrated that sub-pixel resolution techniques and interpolation methods can achieve a measurement precision that is finer than the nominal pixel size when applied to cartilage analysis [21]. While no previous studies by other groups have used the same methodology or threshold criteria for direct comparison, we consider our approach reasonable for assessing variability in the context of fully automatic segmentation and region-level averaging [22]. Nevertheless, the influence of voxel dimensions, and particularly partial volume effects, should be acknowledged when interpreting small measurement differences.

Several limitations of the present study should be noted. First, the MRI scans were performed on the same day by the same technician. If the scans were performed on different days by different technicians, the interscan measurement error might be larger. Second, in the analysis of the distribution of interscan measurement errors, the seven regions of the knee cartilage were analyzed together; however, the error may differ between regions. Third, the number of subjects, at 50, was relatively small. Although the distribution of interscan measurement errors is thought to follow a normal distribution, the sample size in this study may not be sufficient to ensure an adequate fit to a normal distribution. Fourth, only the right knee was analyzed to avoid potential laterality mismatches during data processing. This precaution was taken to minimize errors in side identification during this cross-sectional study involving multiple MRI systems.

In conclusion, the AI-based 3D analysis system developed in this study automatically quantifies knee cartilage thickness from MRI. In the entire cohort (KL1–4), 37%, 67%, and 93% had interscan errors ≤ 0.05 mm, ≤ 0.10 mm, and ≤ 0.20 mm, respectively. This system enables relatively reliable monitoring of cartilage thickness in knee osteoarthritis, and can therefore be used to evaluate the progression of this disease and determine the effectiveness of disease-modifying osteoarthritis drugs.

## Supporting information

S1 Table Dataset. (XLSX)

## Abbreviations:

MRI: magnetic resonance imaging
3D MRI: three-dimensional MRI
2D MRI: two-dimensional MRI
SPGR: spoiled gradient
PDW: proton-density weighted
DICOM: digital imaging and communications in medicine
PMF: posterior medial femoral
PLF: posterior lateral femoral
AMF: anterior medial femoral
ALF: anterior lateral femoral
MT: medial tibial
LT: lateral tibial
P: patellar

## References

[1] Sharma L. Osteoarthritis of the Knee. N Engl J Med. 2021;384(1):51–9. doi: 10.1056/NEJMcp1903768 33406330

[2] Kim JS, Borges S, Clauw DJ, Conaghan PG, Felson DT, Fleming TR, et al. FDA/Arthritis Foundation osteoarthritis drug development workshop recap: Assessment of long-term benefit. Semin Arthritis Rheum. 2022;56:152070. doi: 10.1016/j.semarthrit.2022.152070 35870222 PMC9452453

[3] Aoki H, Ozeki N, Katano H, Hyodo A, Miura Y, Matsuda J, et al. Relationship between medial meniscus extrusion and cartilage measurements in the knee by fully automatic three-dimensional MRI analysis. BMC Musculoskelet Disord. 2020;21(1):742. doi: 10.1186/s12891-020-03768-3 33183257 PMC7664063

[4] Sekiya I, Katano H, Mizuno M, Koga H, Masumoto J, Tomita M, et al. Alterations in cartilage quantification before and after injections of mesenchymal stem cells into osteoarthritic knees. Sci Rep. 2021;11(1):13832. doi: 10.1038/s41598-021-93462-8 34226650 PMC8257723

[5] Sekiya I, Kohno Y, Hyodo A, Katano H, Komori K, Koga H, et al. Interscan measurement error of knee cartilage thickness and projected cartilage area ratio at 9 regions and 45 subregions by fully automatic three-dimensional MRI analysis. Eur J Radiol. 2021;139:109700. doi: 10.1016/j.ejrad.2021.109700 33865065

[6] Katano H, Nagai K, Kaneko H. Variations in knee cartilage thickness: fully automatic three-dimensional analysis of MRIs from five manufacturers. Eur J Radiol. 2024;176:111528. 38815306 10.1016/j.ejrad.2024.111528

[7] Miura Y, Ozeki N, Katano H, Aoki H, Okanouchi N, Tomita M, et al. Difference in the joint space of the medial knee compartment between full extension and Rosenberg weight-bearing radiographs. Eur Radiol. 2022;32(3):1429–37. doi: 10.1007/s00330-021-08253-6 34491384 PMC8831267

[8] Sekiya I, Sasaki S, Miura Y, Aoki H, Katano H, Okanouchi N, et al. Medial tibial osteophyte width strongly reflects medial meniscus extrusion distance and medial joint space width moderately reflects cartilage thickness in knee radiographs. J Magn Reson Imaging. 2022;56(3):824–34. doi: 10.1002/jmri.28079 35084789 PMC9544412

[9] Guermazi A, Hunter DJ, Roemer FW. Plain radiography and magnetic resonance imaging diagnostics in osteoarthritis: validated staging and scoring. J Bone Joint Surg Am. 2009;91 Suppl 1:54–62. doi: 10.2106/JBJS.H.01385 19182026

[10] Sekiya I, Katano H, Guermazi A, Miura Y, Okanouchi N, Tomita M, et al. Association of AI-determined Kellgren-Lawrence grade with medial meniscus extrusion and cartilage thickness by AI-based 3D MRI analysis in early knee osteoarthritis. Sci Rep. 2023;13(1):20093. doi: 10.1038/s41598-023-46953-9 37973855 PMC10654518

[11] Katano H, Ozeki N, Koga H et al. Three-dimensional MRI shows cartilage defect extension with no separation from the meniscus in women in their 70 s with knee osteoarthritis. Sci Rep. 2022;12:4198. 35273291 10.1038/s41598-022-08092-5 PMC8913674

[12] Katano H, Ozeki N, Mizuno M, Endo K, Okanouchi N, Fujita J, et al. Morphological analysis of three-dimensional MR images of patellofemoral joints in asymptomatic subjects. Sci Rep. 2023;13(1):16750. doi: 10.1038/s41598-023-42404-7 37798323 PMC10555988

[13] Disler DG. Articular cartilage in the knee: current MR imaging techniques and applications in clinical practice and research. Invited commentary. Radiographics. 2011;31(1):61–2. doi: 10.1148/Radiographics.31.1.31161 21322827

[14] Gold GE, Chen CA, Koo S, Hargreaves BA, Bangerter NK. Recent advances in MRI of articular cartilage. AJR Am J Roentgenol. 2009;193:628–38. 19696274 10.2214/AJR.09.3042 PMC2879429

[15] Katano H, Kaneko H, Sasaki E, Hashiguchi N, Nagai K, Ishijima M, et al. Same-model and cross-model variability in knee cartilage thickness measurements using 3D MRI systems. PLoS One. 2025;20(6):e0324912. doi: 10.1371/journal.pone.0324912 40512695 PMC12165388

[16] Eckstein F, Charles HC, Buck RJ. Accuracy and precision of quantitative assessment of cartilage morphology by magnetic resonance imaging at 3.0T. Arthritis Rheum. 2005;52:3132–6. 16200592 10.1002/art.21348

[17] Eckstein F, Collins JE, Nevitt MC, Lynch JA, Kraus VB, Katz JN, et al. Brief Report: Cartilage Thickness Change as an Imaging Biomarker of Knee Osteoarthritis Progression: Data From the Foundation for the National Institutes of Health Osteoarthritis Biomarkers Consortium. Arthritis Rheumatol. 2015;67(12):3184–9. doi: 10.1002/art.39324 26316262 PMC5495918

[18] Ciliberti FK, Guerrini L, Gunnarsson AE. CT- and MRI-Based 3D Reconstruction of Knee Joint to Assess Cartilage and Bone. Diagnostics (Basel). 2022;12. 10.3390/diagnostics12020279 PMC8870751 35204370

[19] Guo J, Yan P, Qin Y, Liu M, Ma Y, Li J, et al. Automated measurement and grading of knee cartilage thickness: a deep learning-based approach. Front Med (Lausanne). 2024;11:1337993. doi: 10.3389/fmed.2024.1337993 38487024 PMC10939064

[20] Eckstein F, Cicuttini F, Raynauld J-P, Waterton JC, Peterfy C. Magnetic resonance imaging (MRI) of articular cartilage in knee osteoarthritis (OA): morphological assessment. Osteoarthritis Cartilage. 2006;14 Suppl A:A46-75. doi: 10.1016/j.joca.2006.02.026 16713720

[21] Lee JG, Gumus S, Moon CH, Kwoh CK, Bae KT. Fully automated segmentation of cartilage from the MR images of knee using a multi-atlas and local structural analysis method. Med Phys. 2014;41:092303. 25186408 10.1118/1.4893533 PMC4149695

[22] Badar F, Xia Y. Image interpolation improves the zonal analysis of cartilage T2 relaxation in MRI. Quant Imaging Med Surg. 2017;7(2):227–37. doi: 10.21037/qims.2017.03.04 28516048 PMC5418151

